# Traditional Usage of Wild Fauna among the Local Inhabitants of Ladakh, Trans-Himalayan Region

**DOI:** 10.3390/ani10122317

**Published:** 2020-12-07

**Authors:** Shiekh Marifatul Haq, Eduardo Soares Calixto, Umer Yaqoob, Riyaz Ahmed, Ahmed Hossam Mahmoud, Rainer W. Bussmann, Osama B. Mohammed, Khalid Ahmad, Arshad Mehmood Abbasi

**Affiliations:** 1 Department of Botany, University of Kashmir Srinagar, Jammu and Kashmir 190006, India; snaryan17@gmail.com (S.M.H.); umerraj6668@gmail.com (U.Y.); 2 Wildlife Crime Control Division, Wildlife Trust of India, Noida 201301, India; fo.securehimalayas@wti.org.in; 3 Institute of Biology, University of São Paulo, Ribeirão Preto, São Paulo 05508-060, Brazil; calixtos.edu@gmail.com; 4 Department Zoology, College of Science, King Saud University, P.O. Box 2455, Riyadh 11451, Saudi Arabia; ahmahmoud@ksu.edu.sa (A.H.M.); omohammed@ksu.edu.sa (O.B.M.); 5 Department of Ethnobotany, Institute of Botany, Ilia State University, Tbilisi 0105, Georgia; rbussmann@gmail.com; 6 Department of Environmental Sciences, COMSATS University Islamabad, Abbottabad Campus 22060, Pakistan; khalid.taxonomist@gmail.com

**Keywords:** ethnozoological usage, animal parts, biodiversity hotspot, fauna conservation

## Abstract

**Simple Summary:**

Like plants, animal-based remedies are being utilized traditionally in different cultures around the globe. We explored the traditional usage of wild animals by the local inhabitants in Ladakh area of Trans-Himalayan region, India, using questionnaires and interviews. Furthermore, associations between ethnozoological usage and animal species were also evaluated through multivariate ecological community analysis. The highest priority of local inhabitants was for food followed by medicinal usage. We documented therapeutic uses of 48% of the reported species. Among these *Alectoris chukar* (chukar), *Cuon alpinus* (Asiatic wild dog), *Lepus oiostolus* (hares), *Marmota himalayana* (marmots), *Ovis aries vignei* (Ladakh urial), *Pantholops hodgsonii* (Tibetan antelope), *Procapra picticaudata* (Tibetan gazelle), *Pseudois nayaur* (blue sheep), *Tetraogallus himalayensis* (Himalayan snow), *Tetraogallus tibetanus* (Tibetan snow cock), and *Lutra lutra* (common otter) were reported for the first time from this region and are being used for medicinal purpose. Our findings could be valuable as reference data for policymakers, researchers, land managers, common public, and the other stakeholders to develop logical and scientific approaches for sustainable use of faunal diversity in biodiversity hotspot regions.

**Abstract:**

Zootherapy is accepted all around the globe not only in ancient cultures but different animal derived medicines are also part of the practice in the modern health care systems. The present study assessed the traditional ethnozoological usage of wild animals by local inhabitants in Ladakh region, India, and the reference data for scientific approaches for protection of faunal diversity in trans-Himalayas. The ethnozoological documentation of the animals in Ladakh was carried out through semistructured and close-ended questionnaire surveys and interviews. Multivariate ecological community analysis was used to elucidate the relationship between ethnozoological usage and animal species. Our results showed three animal usage clusters with 32% similarity. Moreover, the similarity in animal usage between digging tools, trophy, handle of tools, decoration, and matting, showed less than 32% of similarity. The highest priority of local people was for food followed by decoration and medicinal usage. The most frequently used animal parts were meat followed by fur and horn. Medicinal uses of 48% of the reported species, i.e., *Alectoris chukar* (chukar), *Cuon alpinus* (Asiatic wild dog), *Lepus oiostolus* (hares), *Marmota himalayana* (marmots), *Ovis aries vignei* (Ladakh urial), *Pantholops hodgsonii* (Tibetan antelope), *Procapra picticaudata* (Tibetan gazelle), *Pseudois nayaur* (blue sheep), *Tetraogallus himalayensis* (Himalayan snow), *Tetraogallus tibetanus* (Tibetan snow cock), and *Lutra lutra* (common otter) were reported for the first time from this region. Our study provides innovative information regarding the ethnozoological knowledge in the Ladakh region and reference data for policymakers, researchers, land managers, common public, and the other stakeholders to develop logical and scientific approaches for sustainable use of faunal diversity in hotspot regions like trans-Himalayas and other similar biodiversity-rich sites.

## 1. Introduction

From the beginning of civilization, the relationship between mankind and animals has been strong and intimate. These intricate relations between mankind and wildlife are found in every culture throughout the world, in numerous types of associations with animals and plants living in their locality [1]. Wild animals play a considerable role in human culture, religion, and economy. The utilization of animals and plants for medicinal purposes has passed in the form of traditional knowledge from generation to generation. Different tribal and ethnic people have been collecting this precious information for generations. While the application of animals in traditional systems of medicine is not new, its documentation is so far very restricted. The exploration and documentation of this indigenous knowledge is essential to get firsthand information about the various uses of animals [1,2].

Zootherapy is accepted all over the globe in ancient cultures [2]. However, it has been found that in modern times uses of animal derived medicines often differ and change. The World Health Organization report stated that out of 252 essential chemicals used in medicines, 8.7 percent have their origin in animals and 11.1 percent in plants [3]. These animal-derived medicines are mainly obtained from animal’s body parts, their metabolic products, or products such as cocoons and nests [4]. Such use is indeed ancient and has long been documented. It is known that Chinese people have been using earthworms for 4000 years to cure various diseases [5]. Further, it has been documented that 1500 animal species are used in Traditional Chinese healthcare [1]. In addition, it has been reported that about 15–20% of the Ayurvedic medicines (traditional Hindu system of medicine) find their origin in animals [6] and about 500 species of invertebrates are being utilized to treat ailments [7]. Chemicals derived from different plants and animals have been used from times immemorial by humans to improve their health [8]. For instance, due to antibacterial, immunological, diuretic, analgesic, anesthetic, and antirheumatic properties, insects make up an important part of modern allopathic medicines [9]. Chitosan, derived from the exoskeleton of insects, is used in modern healthcare systems as an anticoagulant, to reduce blood cholesterol and repair tissues [10]. Similarly, potential anticancer medicines have been obtained from the legs and wings of Taiwanese stag beetles and Asian sulfur butterflies [11]. Many animal species are tested for drug discovery by the pharmaceutical industries. An inhibitor of angiotensin-converting enzyme (ACE) was obtained from snake venom. This ACE enzyme converts the inactive angiotensin into its active form resulting in narrowing of blood vessels and thus increases in blood pressure. However, this inhibitor obtained from snakes prevents the conversion of angiotensin into its active form and thus keeps the pressure at at normal rate [12]. Likewise, several compounds having defensive functions such as alkaloids, steroids, biogenic amines, and peptides have been obtained from amphibians and have numerous pharmacological effects such as myo-toxic, neurotoxic, and cardio-toxic activities [13,14].

Thus, it is imperative to recognize this man–wildlife relationship, and ethno-zoology is a new, attractive research field. However, the cultural and social bonds between wildlife and ethnic people must be taken into consideration and their role is very important in prospecting the medicinal value of the wild animals. The present study assessed the composition, distribution, and traditional ethnozoology usage by local inhabitants in Ladakh region, India. The results of this assessment provide reference data for policymakers, researchers, land managers, common public, and other stakeholders to develop logical and scientific approaches for protection of natural resources and sustainable utilization of fauna diversity in hotspot regions like trans-Himalayas and other similar biodiversity-rich sites.

## 2. Materials and Methods

### 2.1. Study Area

**Figure 1:**
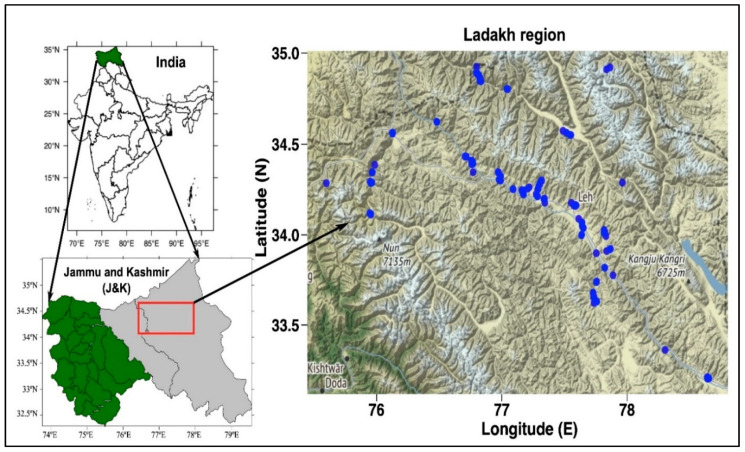
Map of the study area showing the sampling sites (*n* = 54) in the Ladakh region.

Ladakh, the trans-Himalayan region of the Indian Himalaya, is known for its rocky and uneven terrain with extreme cold climate, diverse and rich wild fauna, and alpine flora. It is a region administered by India as a union territory located at 34°12′34.2540″ N and 77°36′54.4032″ E (Figure 1). It is bordered by the Indian state of Himachal Pradesh to the south, Tibet to the east, Jammu and Kashmir and Baltistan to the west, and the southwest corner of Xinjiang across the Karakoram Pass to the far north. It extends from the Siachen Glacier in the Karakoram Range to the north to the main Great Himalayas to the south [15]. In August 2019, a reorganization act was passed by the Government of India containing the provision to remap Ladakh as a union territory [16]. As per 2011, census population of Ladakh is 274,289. This population is divided between Buddhist (77%), Muslim (14%), and Hindus (8%). Ladakhi, also known as Bodhi or Bhoti, is a Tibetic language spoken in Ladakh [17].

The principal crops grown are barley and wheat, while rice was always considered as a luxury item in their diet. Now, with new government subsidies rice has become a cheap staple [18]. The Pashmina goat or Changthangi goat (*Capra aegagrushircus*) is the main source of income from which the famous Pashmina shawls are obtained [15]. Many people in Ladakh were originally associated with textile production, carpets, dyestuffs, and caravan trade between Punjab and Xinjiang. However, currently the Chinese Government has closed the borders and the population in Ladakh suffers [19]. Tourism accounts for about 4% of peoples’ employment but contributes 50% of the GDP to the region [19].

Ladakh receives less than 50 mm precipitation annually, mainly in the form of snow [15]. The soil remains frozen in winter and has low humidity in summer. Animals like sheep and goats are used for food (meat), horses and donkeys for transportation, yaks and cows are used to produce butter, milk, cheeses, and wool, Tzos are used to plough [15].

Ladakh is the best area for snow leopard (Panthera uncia) in India and is also known to have a high diversity of wild sheep and goats. Tibetan argali (*Ovis ammon*), Asiatic wild dog (*Cuon alpinus*), Blue sheep (*Pseudois nayaur*), Asiatic Ibex (*Capra sibirica*), Ladakh Urial (*Ovis aries vignei*), are found here. Ladakh is also home to the endangered Tibetan antelope (*Pantholops hodgsonii*), Tibetan gazelle (*Procapr apicticaudata*), and kiang, or Tibetan wild ass (*Equus kiang*), which are distributed in Changthang area. The Eurasian lynx, Pallas’s cat (*Otocolobus manul*), are other carnivores of Ladakh. Brown bear (*Ursus arctos*), Tibetan wolf, and sand fox are other carnivores distributed in Ladakh. Many bird species include Himalayan snowcock, Tibetan snowcock, and chukar can be commonly seen in Ladakh [20,21]. Ladakh’s flora is a rich repository of medicinal and aromatic plants. The vegetation of the region can be classified into three broad categories—Alpine mesophytes, Oasitic or Riparian vegetation, and arid vegetation [22,23]. 

### 2.2. Survey and Data Collection

**Figure 2:**
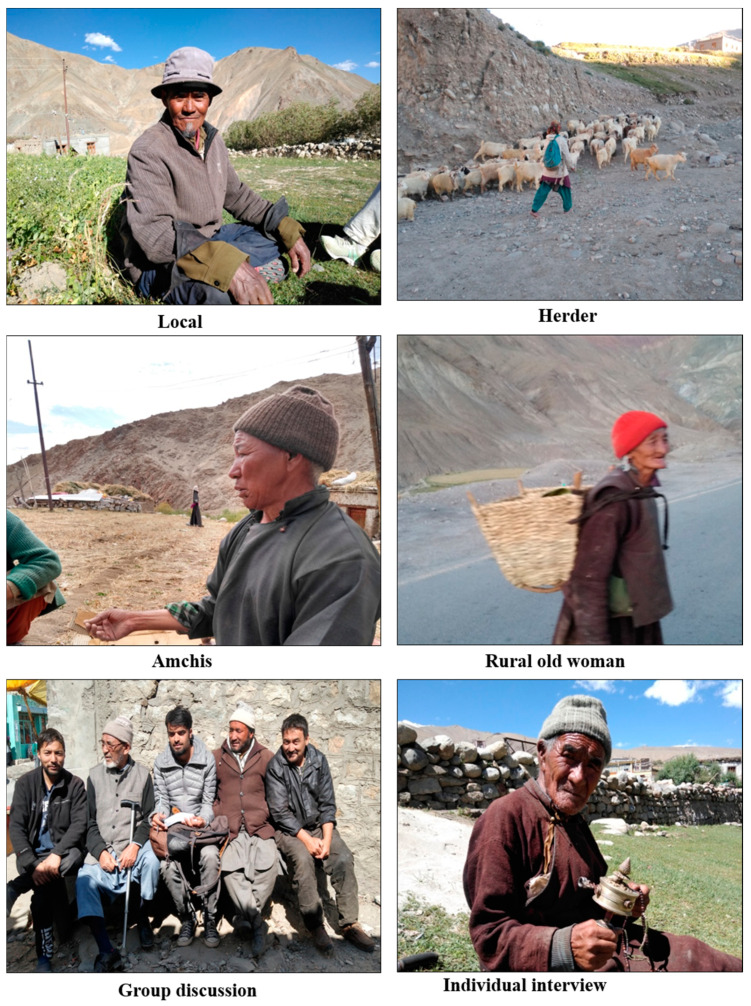
Diverse ethnic groups of the study area.

**Figure 3:**
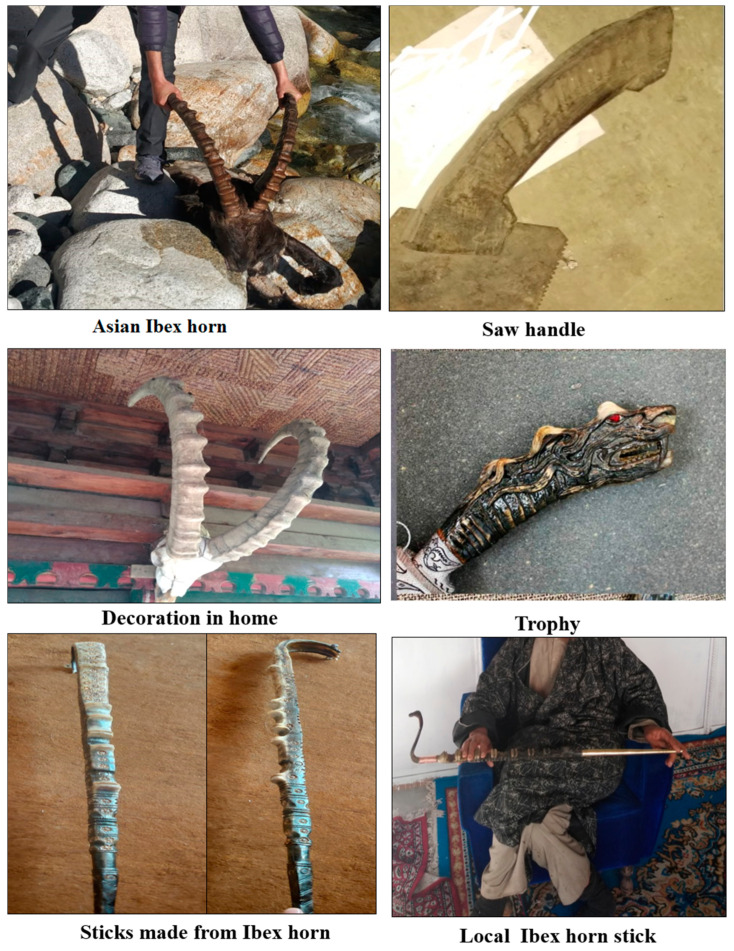
Uses of different part of wild animals.

**Figure 4:**
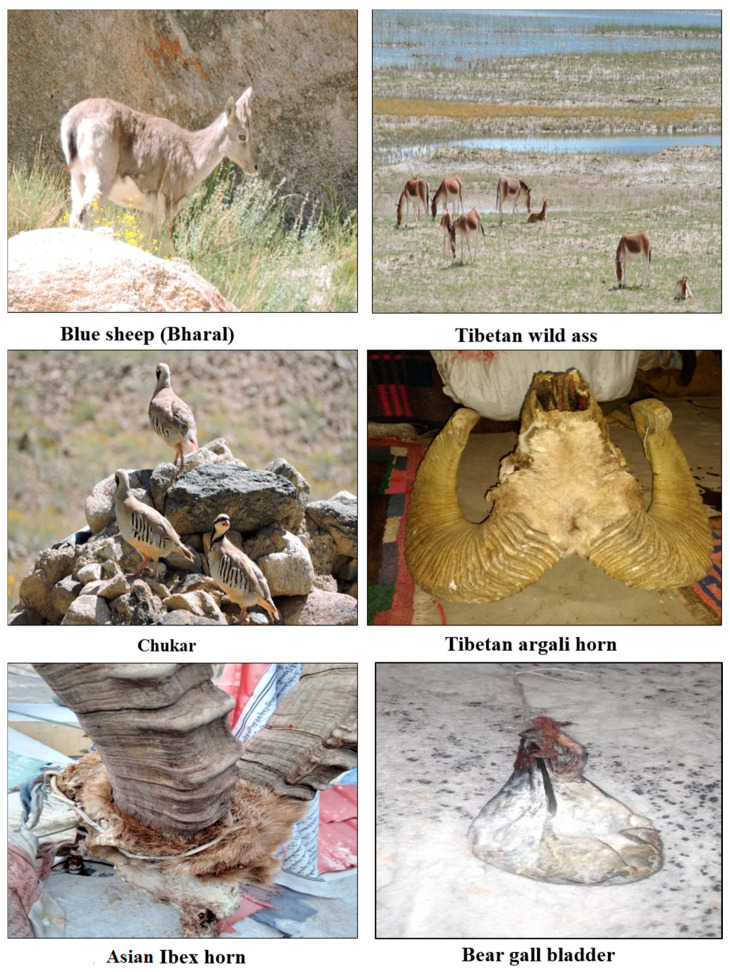
Fauna/parts sited during field study.

First reconnaissance field surveys were carried out to get an understanding about the nature of terrain, accessibility, and distribution of the fauna in the study area. A forest working plan was accessed for authentication of administrative jurisdiction, geographical location, and wild fauna. The selected sites (n = 54) were observed in five field trips during the study year 2019. The methodology was based on interviews (269 interviews, of which 184 were individual based and 17 were group based) using semistructured and closed ended questionnaires and discussions to document the folk knowledge and ethnozoological uses of animal resources. The information was collected from diverse groups of the area, i.e., Amchis (traditional doctors), hunters, herders, shopkeepers, farmers, daily wage labourers, hotel owners, museum owners, housewives, govt. employees etc. by interviewing and completing a questionnaire. To document the traditional knowledge of wild fauna of the study area, questionnaires and interviews method (intersperse fact-based questions) was used. The interviews were carried out both at individual and group level. All interviews were conducted only after obtaining prior informed consent of the village heads, tribal leaders, and individual informants, by explaining clearly the objectives of the study. Participants whose photos are shown in Figure 2, Figure 3 and Figure 4 agreed to this use. The most important ethnic groups are the Amchis, remote, rural or far flung villagers, herders, and hunters, who are directly dependent on wildlife products for their livelihood (Figure 2). A local community member of these tribes who knew the norms and traditions of that indigenous society was taken as a guide 

Informants were asked about the usage of animals, e.g., as medicinal resource, for clothes, digging, trophy, handle, decoration and matting, fun/sport, and food (Figure 3). In addition, the animal part used such as meat, fur, eggs, horns, bones, skin, domesticating, or any other parts such as tongue, heart, and liver (Figure 4).

Field-based personal observations, information from local informers, and both formal and informal discussions were carried out for additional information. The field study was carried out in diverse age-sex groups (young, old, and middle). Further, the informants were asked about perceptions regarding the wildlife. The respondents were further asked about their species preference if they utilized a species for self-consumption or trade for earning purposes.

### 2.3. Data Analysis

Animal data were statistically analyzed to find the relationship between ethnozoological usage and animal species. The presence/absence data were subjected to the classification of different ethnozoological similarities and differences among the different animal usage via PAST software [24,25]. Using this method, more similar groups come close to each other and dissimilar groups are shown as distant in the cluster from each other. In other words, if an animal species has two or more different usages, these usages will be clustered closer. Heatmap and Sørensen’s (Bray–Curtis) distance was used to identify significant differences among the different ethnozoological usage similarities [26,27]. Principal components analysis (PCA) was used to find hypothetical variables (components) that account for as much of the variance in our multidimensional data as possible. The contribution of different animal part usage was displayed in chord diagrams using circlize package [28] in R software 4.0.0 [29]. To run a preference analysis, i.e., whether there is difference between animal usage, and between animal parts used, we used a Generalized Linear Model (GLM) with binomial distribution followed by Likelihood-Ratio test using the packages “stats” [30] and “car” [31]. For that, we used the number of species divided by the total number of species observed multiplied by 100.

## 3. Results and Discussion

Information about the utilization of wild as well as domestic fauna served as leads for the bioprospecting of various medicinal drugs as well as other commercially valuable compounds. From early times, people living around wild habitats have been using animals for a large variety of purposes. Ladakh (trans-Himalayan region) is known for its rich alpine medicinal plant wealth and wild fauna [32].

### 3.1. Local Respondents and Their Perceptions about Wildlife

The interviewees represented a diverse array of ethnic groups in the area (Table 1). Among the 269 respondents, 208 (77.3%) were men and 61 (22.7%) were women. The largest proportion of the informants was elderly, i.e., above 45 years old (88%). A major part of respondents (65%) was illiterate. The age of the respondents ranged between 25 and 88 years. Most of the respondents were 46–65 (48%) years old, followed by 66–88 (40%), and 25–45 (12%). The respondents interviewed included farmers (30%), housewives (19%), herders (9%), Govt. employees (8%), daily wage labourors (8%), hunters (6%), hotel owners (6%), shopkeepers (5%), and museum owners (1%). About 66% informants were Buddhists, while the other 34% were Muslims. Among these different groups, the Amchis, hunters, herders/shepherds, and those inhabiting rural and/or far-flung areas are much more informative as compared to others as they are directly dependent on animals’ products for their livelihoods. Many faunal species had cultural values. Different mammalian and bird species were used in magic or rituals. The bones, meat, and hair of *Panthera uncia* and *Camelus bactrianus* (camel) were used in the treatment of black magic (Kalaa Jadoo). Similarly, the horns of *Ovis aries vignei* and *Capra sibirica* were used as trophies at the entrance of temples and houses to protect the families from bad spirits. Likewise, the horns and bones of *Capra sibirica* were use as defensive and digging tools. Similarly, the bones of the Brown bear were used as defensive tools. The dried meat of *Lynx lynx* and *Panthera uncia* were used as amulets to protect the body from diseases and masculine disorders. Furthermore, it was also found that decorated *Camelus bactrianus* were used in wedding ceremonies. In addition, Ibex horn, brown bear head, and fox tail were used for decoration purposes. The wool of the Tibetan antelope is known for making famous Shahtoosh and as a status symbol.

As there are different religious communities in the Ladakh province, these different religious communities are diverse in terms of cultural values. It was found that Muslims ate only certain bird and animal species as they follow the teachings of Islam. Muslim communities usually preferred hunting particular bird and mammalian species for meat, but Buddhists may collect and eat the meat of dead animals already killed by an apex predator such as snow leopard, bear, and wolf or by natural death. We observed that local Amchis and old people had great familiarities with traditional usage of animal species, compared to younger participants; and this trend was comparable to previous reports from other parts of Himalayas [33,34,35,36,37].

The culture-specific sets of interwoven beliefs and practices need to be carefully evaluated in a holistic framework [38], because it affects food and nutrition and health of indigenous people in multiple ways. We also evaluated the perception of locals towards wildlife. Three classes of people were identified:

1. The majority (54%) of participants considered wildlife as part of the ecosystem and culture and had keen belief that wildlife was to be conserved and protected.
2. A second class (36%) observed wildlife as a threat to humans and agriculture, and,
3. A small minority (10%) had no preconceived ideas.

Concerning the second class (36%), which regarded wildlife as a threat, further studies were carried out regarding the number of domestic animals lost to wild animals per year. Our findings revealed that 5.8 ± 2.68 individuals per year (range between 2 and 17 organisms) were lost, including sheep, goats, and other large animals, hence resulting in human-wildlife conflict. To mitigate these problems, locals take advantage of pet dogs, campfires, and sometimes professional hunters are also called. Rigg et al. [37] while studying carnivore-livestock conflicts in Europe (Slovakia) suggested that livestock guarding dogs are particularly appropriate for wolfs, which cause four to six times more damage to domestic and wild animals than brown bear (*Ursus arctos*) [37]. In the meantime, lack of motivation and awareness are some obstacles.

### 3.2. Animal Usage Classification

**Figure 5:**
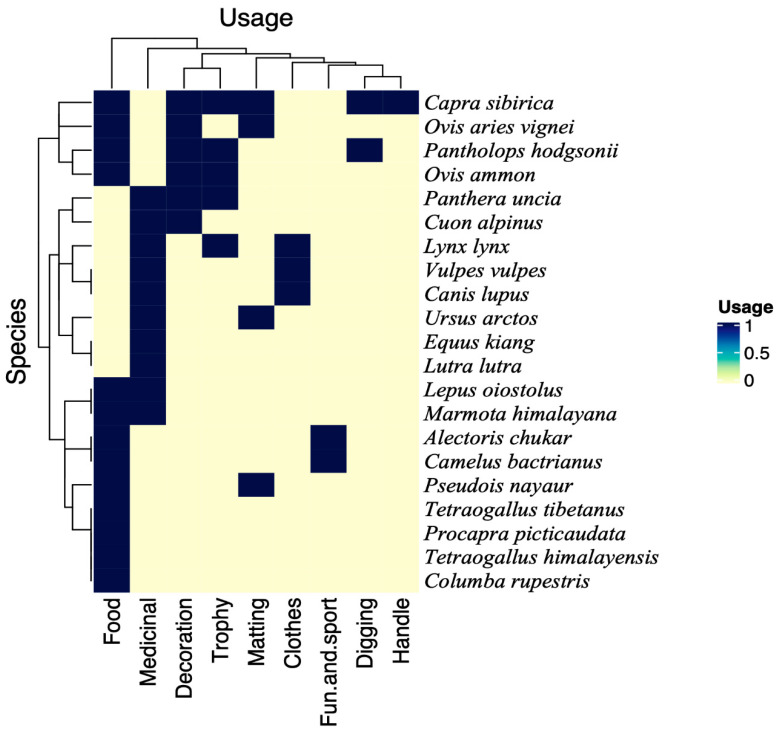
Cluster diagram of the fauna based on animal usage in Ladakh.

**Figure 6:**
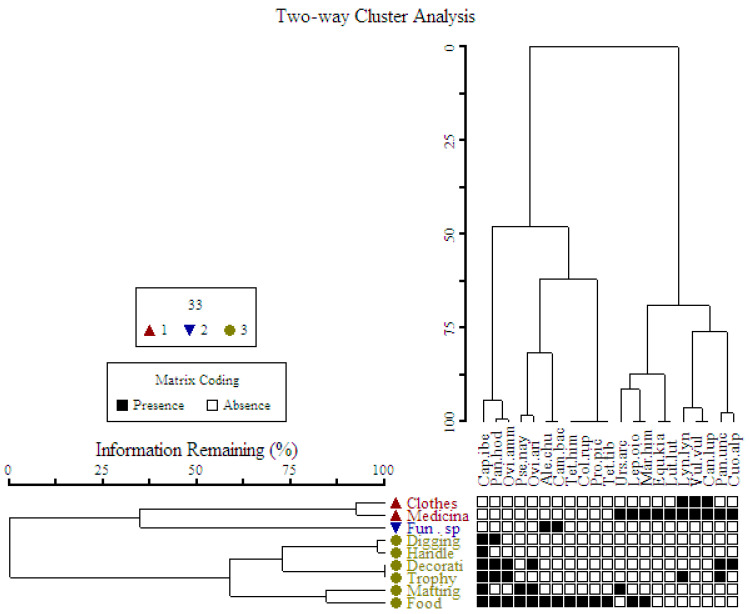
Two Way Cluster Analysis based on Sørenson’s similarity index of animal species.

The respective usage of animal species and ways of use are present in Table 2. Each animal species found in the region is worth mentioning, but a few of them had key significance in the traditional usage. The heatmap uses presence/absence data to show the species usage, and the clustering analysis will cluster usages that share the same species. The wild fauna in the present study showed considerable variation in usage (Table 2, Figure 5). We observed that most of the animals (e.g., *Panthera uncia* (Snow leopard), *Canis lupus* (Tibetan wolf), *Lynx lynx* (Eurasian lynx), *Cuon alpinus* (Asiatic wild dog), *Ursus arctos* (Brown bear), *Equus kiang* (Wild ass) and *Vulpes vulpes* (Tibetan sand fox) were of medicinal use. Few animal species such as *Capra sibirica* (Asiatic Ibex), *Ovis aries vignei* (Ladakh Urial), *Pantholops hodgsonii* (Tibetan antelope), and *Ovis ammon* (Tibetan argali) were used for food as well as other ethnozoological usage (trophy and decoration) (Table 1, Figure 5). The clustering of wild fauna based on the ethnozoological usage is presented in Figure 5 where animals grouped in one limb are more similar in usages and show proximity to each other. The Two-Way Cluster Analyses of nine ethnozoological usage including 21 animal species results in four major clusters (Figure 6). In diagram the zero-show absence whereas the numbers indicate the presence of animal species in the particular ethnozoological usage cluster. The analyses classified trans-Himalayas fauna into four groups that were recognized on the basis of indicator species, i.e., group one with *Tetraogallus tibetanus* (Tibetan snow cock), *Columba rupestris* (Pigeon), *Procapra picticaudata* (Tibetan gazelle), *Marmota himalayana* (Marmots), *Tetraogallus himalayensis* (Himalayan snow), *Lepus oiostolus* (Hares), *Camelus bactrianus* (Double-hump) and *Alectoris chukar* (Chukar). Group two with *Ovis aries vignei* (Ladakh Urial), *Ovis ammon* (Tibetan argali), *Pseudois nayaur* (Blue sheep), *Capra sibirica* (Asiatic Ibex), and *Pantholops hodgsonii* (Tibetan antelope). Group three including species *Panthera uncia* (Snow leopard), *Canis lupus* (Tibetan wolf), and *Lynx lynx* (Eurasian lynx). Group four with *Ursus arctos* (Brown bear), *Lutra lutra* (Common Otter), *Equus kiang* (Wild ass), and *Vulpes vulpes* (Tibetan sand fox). Similar classifications were also carried out by previous ethnozoological workers from Pakistan Himalayas [34,35].

### 3.3. Preference Analysis

**Figure 7:**
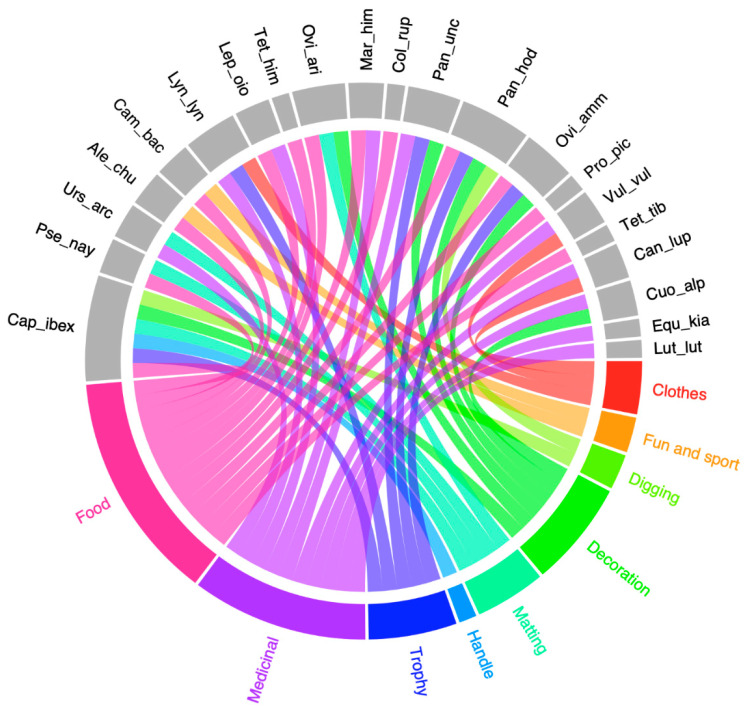
Preference analysis of animal species distribution according to animal usage in Ladakh trans-Himalayan region, India. Full animal names are depicted in Table 1.

**Figure 8:**
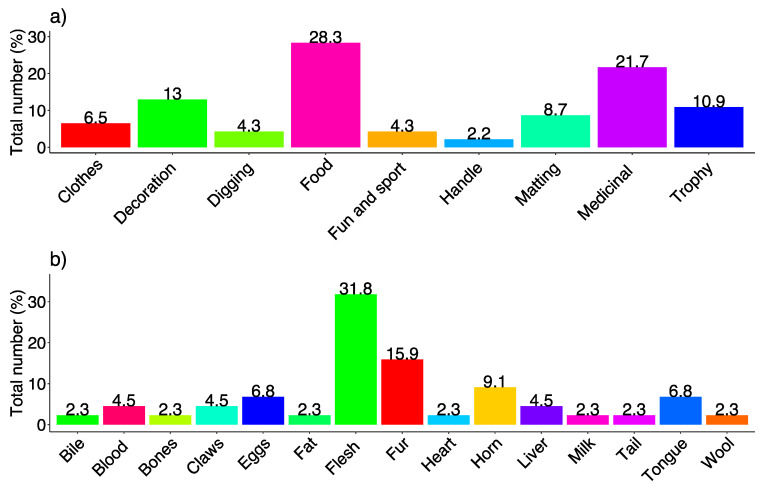
(**a**) Percentage of different ethnozoological usage; (**b**). Percentage of different animal body parts used. The percentage is based on the number of species used divided by the total number of species multiplied by 100.

**Figure 9:**
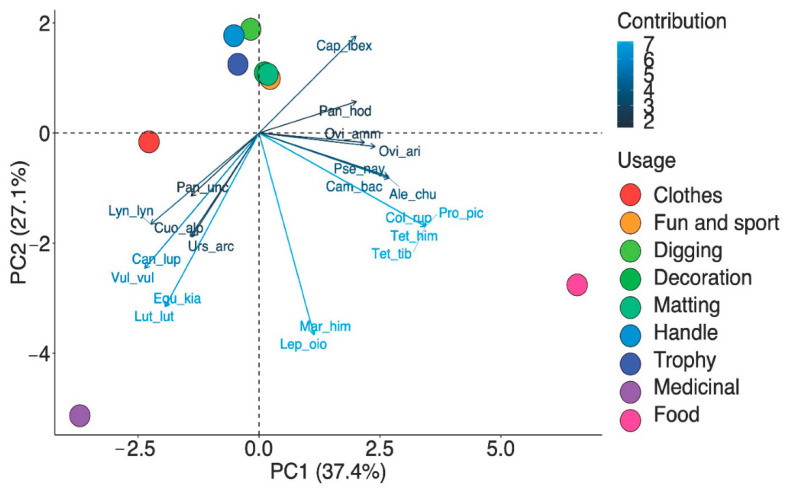
Principal Component Analyses (PCA) biplot of different provisioning services in Ladakh.

From the obtained data, it is clear that the fauna of Ladakh has food, medicinal, aesthetic, and agriculture values. Differences in animal usage based on food, medicine, decoration, clothes/trophy, and digging/fun/sports were observed (Figure 7). The results of preference analysis showed a significant difference (χ^2^ = 32.652, df = 8, *p* < 0.001) in animal usage. The highest priority of local people was for food (28%), followed by medicinal (22%), decoration (13%), trophy (11%), matting (9%), clothes (7%), fun and sport, and digging (4% each) as shown in Figure 8a. This fact is also supported by PCA which showed distinct usage segregation based on variations in the preference levels (Figure 9). Previous ethnozoological studies have also documented that animals have a close relationship with the mankind, providing food, medicine, and clothes [39,40].

**Figure 10:**
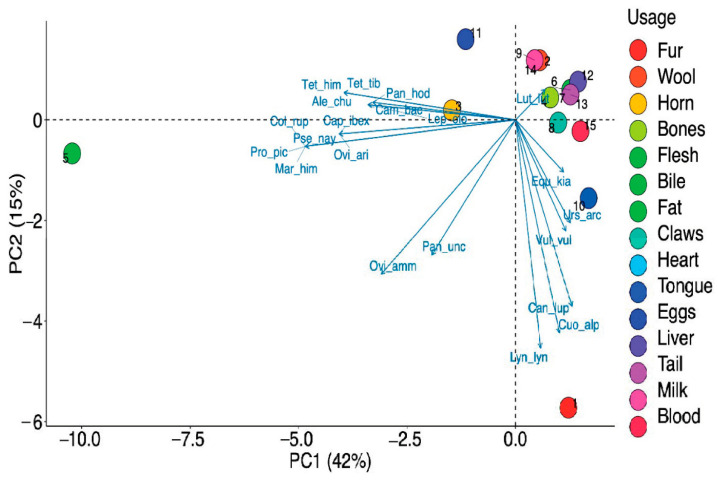
Principal Component Analyses (PCA) biplot of different part(s) usage.

We emphasized the various parts of the animals utilized along with their use for various purposes. Different parts of animals were documented for traditional usage with a significant difference (χ^2^ = 50.486, df = 14, *p* < 0.001) between their usages. Among the various parts of animals (Figure 8b), meat was the most utilized with 31% of usage. Meat is one of the most vital sources of protein in such rural areas [38]. Fur was the next body part of animals most prominently used with 17% of usage. The other parts used were horns (9%), tongue, blood and eggs (7% each), claws and liver (4% each), bile, fat, bone, heart, milk, and wool (2% each) (Figure 8b). This fact is also supported by PCA which showed distinct usage segregation based on variations in the preference levels (Figure 10).

### 3.4. Medicinal Uses of Animal Species 

The local inhabitants of the study possessed significant traditional knowledge and used different animals to treat various health disorders. In the present study we reported 21 animal species traditionally used by the inhabitants of Ladakh as medicine (Table 2). To the best of our knowledge, medicinal uses of 48% of the reported species, i.e., *Alectoris chukar* (Chukar), *Cuon alpinus* (Asiatic wild dog), *Lepus oiostolus* (Hares), *Marmota himalayana* (Marmots), *Ovis aries vignei* (Ladakh Urial), *Pantholops hodgsonii* (Tibetan antelope), *Procapra picticaudata* (Tibetan gazelle), *Pseudois nayaur* (Blue sheep), *Tetraogallus himalayensis* (Himalayan snow), *Tetraogallus tibetanus* (Tibetan snow cock), and *Lutra lutra* (Common Otter) have rarely been reported before. Likewise, for the rest of the species variations in part(s) used, mode of preparation, and diseases treated were also noted (Table 2). Such disparities in the medicinal uses of animal species reported form Ladakh and other areas revealed cross culture differences in traditional knowledge. As knowledge of indigenous communities on bioresource utilization depends on their perceptions, source or origin of knowledge, and mode of interactions with surrounding environment.

For instance, inhabitants of the Ladakh region use tongue, stomach, and blood of *Canis lupus* (Tibetan wolf/Shanku), against inflammation, to increase digestion and for the treatment of diabetes, respectively. However, present uses were different than previously documented by Alves et al. [42], who reported *C. lupus* against various infectious diseases, asthma, and menstrual cramps. Similarly, Bile and fat of *Ursus arctos* (Brown bear/Denmo) are used to treat pulmonary affliction and treatment of bone and joint pain. Alves et al. [46] reported that body parts of same species effective in convulsion, improving eyesight, reducing pain and inflammation, as well as fever. Meat of *Camelus bactrianus* (Double-hump camel/Nabong) is used to gain body strength and vitality, and its milk is a sexual stimulant and antidote to various insect poisons. These uses of *C. bactrianus* were found to be different compared to previous reports [34,41]. Likewise, use of *Columba rupestris* (Pigeon/Mukron) against inflammation was different than uses reported by Yeshi et al. [44]. Ladakhi communities use bile and dried meat of *Panthera uncia* to treat respiratory disorders and body weakness, whereas nails and hair of the same species were reported to keep away the evil spirits [48]. Similarly, ethnomedicinal uses of *Vulpes vulpes* (Tibetan sand fox/Watse) and *Equus kiang* (Wild ass/Khyang and Gorkhar) as given in Table 2, were different from previous reports [43,45]. The decoction of horns (crushed) of *Capra sibirica* is used against chest infections in the studied area. This use was similar as reported previously [49]. In the Ladakh region, the use of *Lynx lynx* dried meat to overcome body weakness and arthritis was found similar to that reported by Alves et al. [46]. To take full advantage of the potential benefits of traditional animal-based medicines, we need integration of traditional and biomedical medicine and health care [35]. Closer combination may advance the quality, effectiveness, and safety of traditional medicinal services and may at the same time enrich the quality, knowledge, and cultural analogies of diverse medical care services [35].

### 3.5. Wildlife Diversity and Threats

Among the carnivorous animals, the most common species were: Snow leopard (*Panthera uncia)*, Brown bear (*Ursus arctos*), Eurasian lynx (*Lynx lynx*), Asiatic wild dog (*Cuon alpinus*), and Tibetan wolf (*Canis lupus chanco*). In addition, other species found were: Tibetan argali, Blue sheep, Asiatic ibex, and Ladakhi urial. The endangered snow leopard (*Panthera uncia*) is a large cat endemic to the mountain of this region. The snow leopard’s main prey species are the Tibetan argali (*Ovis ammon*), Asiatic wild dog (*Cuon alpinus*), Blue sheep (*Pseudois nayaur*), Asiatic Ibex (*Capra sibirica*), Ladakh Urial (*Ovis aries vignei*), and Tibetan wolf (*Canis lupus chanco*). Ladakh is also home to the endangered Tibetan antelope (*Pantholo pshodgsonii*), known as chiru in Indian English or Ladakhi tsos, and Tibetan gazelle (*Procapra picticaudata*), which is common in the grasslands of Changthang area. Another rare cat that preys on smaller herbivores in Ladakh is the Eurasian lynx. Brown bear (*Ursus arctos*) are found in the Suru valley and the area around Dras. The other smaller animals, marmots, hares, and several types of pika and vole are common. The presence of these animals (mammals) was also reported by Sharma [32]. However, we also documented some species of birds like Himalayan snow cock, Tibetan snow cock, and chukar with ethnozoological usage [32]. An estimated 5.8 individuals (ranges between 2 and 17 organisms), of livestock, i.e., sheep, goats and large sized animals are lost by local inhabitants per annum due to wild carnivorous attacks. The higher part of the range comprises of the herder community. Local inhabitants protect livestock from wild carnivorous by keeping guarding dogs, followed by campfire, and sometimes by professional hunters. 

Our study revealed that traditional knowledge has not only a considerable pharmacological role but is also linked with various cultural beliefs and customs of aboriginals. This study acts as a base to demonstrate the scientific confirmation of therapeutic efficiency of different animal based traditional drugs utilized by the indigenous people and might allow for the discovery of some novel biocompounds and new drugs. This study also provides insights about the perception of indigenous people about the wildlife and how their perception can be changed by providing awareness about the role of wildlife in sustenance of ecosystems. However, it was found that utilization of these wild animals is known to spread many diseases. These animals are known to contain various viral and bacterial pathogens and their use and trade is known to spread a large number of diseases. Recently, the human population is witnessing one of the worst pandemics the world has ever witnessed, which now is known to have spread from bats. This pandemic caused by the virus SARS CoV-2 started in December 2019 in Wuhan province of China and was declared as global health emergency by WHO [50,51]. These types of diseases spread by these wild animals can be prevented by preventing the illegal trade of many wild animals, especially the wild animals in the threatened categories. In addition, this study provides new insights about the importance of biodiversity in these biodiversity hotspots and offers new mitigation and conservation strategies to be taken for the restoration and preservation of wildlife in Himalayan biodiversity hotspot. However, it is imperative to prevent the change in population dynamics of these wild animals by conservation education and awareness involving all the stakeholders and local ethnic people. Nevertheless, it should also be noted that while framing any action plans for conservation, the local traditions should be respected, otherwise it will do more harm than good [52,53].

## 4. Conclusions 

This study offers new contributions to the knowledge of faunal composition and usage by local inhabitants in the Ladakh mountainous regions. Studying the fauna composition and usage of wild animals is fundamental to understanding the association between mountainous communities and animal sources of the region and how the animal population in these regions is being affected by human actions. The use of these animals’ products for different purposes becomes more and more vital for the people of this biodiversity hotspot region. This study will not only make the young generation more aware about their traditional knowledge related to uses of animals and their parts but also the people will have easy and cheap remedies which they think will cure some minor diseases. However, careless and improper utilization of this faunal biodiversity is known to radically alter their population and hence affect the ecosystem stability. Thus, we highlight the importance of our study as a tool that will help in the understanding of the faunal diversity in these regions and how conservation and mitigation measures can be put into action for the preservation of the Himalayan wildlife.

## Figures and Tables

**Table 1:** Details of respondents interviewed in the present study.

	Groups	Participants
Respondents Interviewed	Shopkeepers	13
	Farmers	82
	Daily wage laborer	21
	Hotel owners	15
	Museum owners	2
	Housewives	52
	Govt. employees	23
	Amchis	21
	Herders	24
	Hunters	16
Age group	25–45	33
	46–65	128
	65–88	106
Gender	Male	208
	Female	61
Education qualification	5th grade pass	37
	8th grade pass	21
	10th grade pass	15
	12th grade pass	14
	Graduate and above	8
	Illiterate	174
Religion	Buddhist	178
Number	Muslim	91

**Table 2:** Animal species recorded and their ethno-zoological usage in Ladakh region, Trans Himalayas, India.

Scientific Name	English and Local Name	Class Order Family	Traditional Uses	Medicinal Uses	Previous Use with Reference
*Alectoris chukar* J. E. Gray, 1830	Chukar Srakpa	Aves Galliformes Phasianidae	Food (meat and eggs), Fun and Sports.	Meat soup is used for the treatment of paralysis	
*Camelus bactrianus* Linnaeus, 1758	Double-hump camel Nabong	Mammalia Artiodactyla Camelidae	Food (meat), Fun and Sports. Used to treat black magic	Meat is used to gain body strength and vitality, also to relieve joint pain. Milk is used as sexual stimulant and antidote.	Meat stew is used to strengthen bones, relieve arthritis, and stiff limbs. Stomach is used to aid digestion, cure liver disease. Hump is believed to contain Qu tonic that softens human skin [34,41]
*Canis lupus* Linnaeus, 1758	Tibetan wolf Shanku	Mammalia Carnivora Canidae	Medicine (TML), Clothing (fur is used for caps and gloves).	Tongue, stomach, and blood are used to cure inflammation, to increases digestion and treat diabetes, respectively.	Used to cure chicken pox, smallpox, mumps, varicella, asthma, measles, warts, menstrual cramps [42]
*Capra sibirica* Pallas, 1776	Asiatic Ibex Skin	Mammalia Artiodactyla Bovidae	Food, Agriculture, Aesthetic (horn used for making handles, trophy).	Horns are crushed into powder form mixed with hot water and used against chest infections	Horns are used in traditional medicine [43]
*Columba rupestris* Pallas, 1811	Pigeon Mukron	Aves Columbiformes Columbidae	Food (meat), Medicine (TML).	Dropping are used against inflammation.	Flesh gives physical strength and excreta dries away pus and heal swellings [44]
*Cuon alpinus* Pallas, 1811	Asiatic wild dog Phara	Mammalia Carnivora Canidae	Clothing (fur is used in caps and gloves), Medicine (TML).	Tongue is used to cure ulcers	
*Equus kiang* Moorcroft, 1841	Wild ass Khyang and Gorkhar	Mammalia Perissodactyla Equidae	Medicine, transport	Blood is used in rheumatism and eye disorders. And tongue is used in diarrhea.	Penis is used to enhance the men’s virility [45]
*Lepus oiostolus* Hodgson, 1840	Hares Ribong	Mammalia Lagomorpha Leporidae	Food, Medicine (TML).	Dropping are used to treat skin diseases	
*Lutra lutra* Linnaeus, 1758	Common Otter Eurasian Otter Chusham	Mammalia Carnivora Mustelidae	Medicine (TML)	Liver is used against reproductive disorders	
*Lynx lynx* Linnaeus, 1758	Eurasian lynx Yie	Mammalia Carnivora Felidae	Aesthetic (fur), Medicine (TML).	Dried meat is used to overcome body weakness, arthritis.	Used against evil eye’’, arthritis, avoid acne, distend, earache, fever, leishmaniosis, making the child talk; pain in bones, rheumatism, scare, stomachache, wounds [46].
*Marmota himalayana* Hodgson, 1841	Marmots Phia	Mammalia Rodentia Sciuridae	Food, Medicine (TML).	Liver is used to treat bone disorders.	
*Ovis ammon* Linnaeus, 1758	Tibetan argali Nyan	Mammalia Artiodactyla Bovidae	Food (meat), Aesthetic (Horns).	Meat is used to overcome protein deficiency in adults	Hunted for their meat and their horns for CTM [47]
*Ovis aries vignei* Blyth, 1841	Ladakh Urial Shapo	Mammalia Artiodactyla Bovidae	Food (meat), Aesthetic (Horns for homes and shrines).	Meat is used in cough.	
*Panthera uncia* Schreber, 1775	Snow leopard Shan	Mammalia Carnivora Felidae	Social cultural (meat used to protect young ones from black magic), Economic (bones, claws and fur mostly used as a source of income).	Bile is used to treat respiratory disorders; Dry meat is made into amulet to treat body weakness	Nails and Hairs are used to keep away evil spirits [48]
*Pantholops hodgsonii* Abel, 1826	Tibetan antelope Szos	Mammalia Artiodactyla Bovidae	Clothing (Wool is used for making famous “Shahtoosh”). Aesthetic (Horns), Agriculture (Horns)	Horn is used in childbirth	
*Procapra picticaudata* Hodgson, 1846	Tibetan gazelle Goa	Mammalia Artiodactyla Bovidae	Food (meat), Aesthetic (Horns).	Horn is used to treat diarrhea	
*Pseudois nayaur* Hodgson, 1833	Blue sheep Napo	Mammalia Artiodactyla Bovidae	Food (meat), Aesthetic (Horns), Matting (fur)	Horn is used as an antibiotic. And hair is used as poisoning agent.	
*Tetraogallus himalayensis* G. R. Gray, 1843	Himalayan snow cock Ripja	Aves Galliformes Phasianidae	Food (meat), Sometimes domesticated for “fun and sport” or source for eggs.	Meat is used against asthma and cough in children	
*Tetraogallus tibetanus* Gould, 1854	Tibetan snow cock Ticok	Aves Galliformes Phasianidae	Food (meat), Sometimes domesticated for “fun and sport” or source for eggs.	Meat soup is used in the treatment of paralysis. And droppings against inflammation.	
*Ursus arctos* Linnaeus, 1758	Brown bear Denmo	Mammalia Carnivora Ursidae	Medicine (bile and fat), and fur for matting and Aesthetic purposes.	Bile is used to treat pulmonary affliction. Fat is used for treatment of bone and joint pain.	Used to treat liver problem, to improve eyesight in fever fighting, inflammation, swelling and pain reduction. It was also used in the cure of carbuncle of heat type, pyocutaneous diseases and epilepsy [46].
*Vulpes vulpes* Linnaeus, 1758	Tibetan sand fox Watse	Mammalia Carnivora Canidae	Medicine (TML), Clothing (fur is used in caps and gloves).	Lungs are used in lung ulcer, meat is used to over back pain, and rheumatic pain.	Oil is obtained and used against jaundice [43]

## References

[1] Alves R.R.N., Rosa I.L. (2005). Why study the use of animal products in traditional medicines?. J. Ethnobiol. Ethnomed..

[2] Lohani U. (2010). Man-animal relationships in Central Nepal. J. Ethnobiol. Ethnomed..

[3] Marques J.G. (1997). Fauna medicinal: Recurso do ambiente ou ameaça à biodiversidade. Mutum.

[4] Costa-Neto E.M., Marques J.G. (2000). Conhecimento ictiológico tradicional ea distribuição temporal e espacial de recursos pesqueiros pelos pescadores de Conde, Estado da Bahia, Brasil. Etnoecológica.

[5] Zhang Z., Wang F. (1992). Effects of crude extract of earthworm on promoting blood circulation to removing stasis. Soil Biol. Biochem..

[6] Unnikrishnan P. (1998). Animals in Ayurveda. Amruth Suppl..

[7] Oudhia P. (1995). Traditional knowledge about medicinal insects, mites and spiders in Chhattisgarh, India. Insect Environ..

[8] Beetz A., Uvnäs-Moberg K., Julius H., Kotrschal K. (2012). Psychosocial and psychophysiological effects of human-animal interactions: The possible role of oxytocin. Front. Psychol..

[9] Yamakawa M. (1998). Insect antibacterial proteins. J. Sericult. Sci. Jpn..

[10] Goodman W.G. (1989). Chitin: A magic bullet. Food Insect News.

[11] Kunin W., Lawton J.H., Kunin A.A., Lawton H. (1996). Does biodiversity matter? Evaluating the case for conserving species. Biodivers. Biol. Numbers Differ..

[12] Bisset N.G. (1991). One man’s poison, another man’s medicine. J. Ethnobiol..

[13] Daly J.W. (2003). Amphibian skin: A remarkable source of biologically active arthropod alkaloids. J. Med. Chem..

[14] Clarke B.T. (1997). The natural history of amphibian skin secretions, their normal functioning and potential medical applications. Biol. Rev..

[15] Pant S., Rinchen T., Butola J.S. (2018). Indigenous knowledge on bio-resources management for sustainable livelihood by the cold desert people, Trans-Himalaya, Ladakh, India. Ind. J. Nat. Prod. Resour..

[16] (2019). The Jammu and Kashmir Reorganisation Bill. http://www.prsindia.org/sites/default/files/bill_files/Jammu%20and%20Kashmir%20Reorganisation%20Bill%2C%202019.pdf.

[17] Rizvi J. (1999). Ladakh: Crossroads of High Asia.

[18] Bray J. (1991). Ladakhi history and Indian nationhood. South Asia Res..

[19] Rajashekariah K., Chandan P. (2013). Value Chain Mapping of Tourism in Ladakh.

[20] Fox J.L., Nurbu C., Chundawat R.S. (1991). The mountain ungulates of Ladakh, India. Biol. Conserv..

[21] Namgail T. (2009). Mountain ungulates of the Trans-Himalayan region of Ladakh, India. Int. J. Wilderness.

[22] Rawat G.S., Adhikari B.S. (2005). Floristics and distribution of plant communities across moisture and topographic gradients in Tso Kar basin, Changthang plateau, eastern Ladakh. Arct. Antarct. Alp. Res..

[23] Stewart R.R. (1916). The flora of Ladak, Western Tibet. II. List of Ladak plants. Bull. Torrey Bot..

[24] Greig-Smith P. (2010). Quantitative Plant Ecology.

[25] Hammer O., Harper D.A.T., Ryan P.D. (2001). PAST: Paleontological statistics software package for education and data analysis. Palaeontol. Electron..

[26] Sorensen T.A. (1948). A method of establishing groups of equal amplitude in plant sociology based on similarity of species content and its application to analyses of the vegetation on Danish commons. Vidensk. Selsk. Biol. Skr..

[27] Dalirsefat S.B., Da Silva Meyer A., Mirhoseini S.Z. (2009). Comparison of similarity coefficients used for cluster analysis with amplified fragment length polymorphism markers in the silkworm Bombyx mori. J. Insect Sci..

[28] Gu Z., Gu L., Eils R., Schlesner M., Brors B. (2014). Circlize implements and enhances circular visualization in R. Bioinformatics.

[29] R Core Team (2018). The R Project for Statistical Computing. http://www.R-project.org.

[30] R Core Team (2020). R: A Language and Environment for Statistical Computing.

[31] Fox J., Weisberg S. (2011). An {R} Companion to Applied Regression.

[32] Sharma I. (2017). Mammal’s diversity of Ladakh (Jammu and Kashmir), India. Int. J. Fauna Biol..

[33] Betlu A.L.S. (2017). Indigenous knowledge of zootherapeutic use among the Biate tribe of Dima Hasao District, Assam, Northeastern India. J. Ethnobiol. Ethnomed..

[34] Altaf M. (2016). Assessment of Avian and Mammalian Diversity at Selected Sites along River Chenab in Wildlife and Ecology.

[35] Altaf M., Umair M., Abbasi A.R., Muhammad N., Abbasi A.M. (2018). Ethnomedicinal applications of animal species by the local communities of Punjab, Pakistan. J. Ethnobiol. Ethnomed..

[36] Alonso E.B. (2015). The Impact of Culture, Religion and Traditional Knowledge on Food and Nutrition Security in Developing Countries, No. 2201-2019-1458.

[37] Rigg R., Fino S., Wechselberger M., Gorman M.L., Sillero-Zubiri C., Macdonald D.W. (2011). Mitigating carnivore–livestock conflict in Europe: Lessons from Slovakia. Oryx.

[38] Kakati L.N., Doulo V. (2002). Indigenous knowledge system of zootherapeutic use by Chakhesang tribe of Nagaland, India. J. Hum. Ecol..

[39] Kruuk S., Kruuk H. (2002). Hunter and Hunted: Relationships between Carnivores and People.

[40] Alves R.R.N., Rosa I.L., Santana G.G. (2007). The role of animal-derived remedies as complementary medicine in Brazil. BioScience.

[41] Kadim I.T., Mahgoub O., Mbaga M. (2014). Potential of camel meat as a non-traditional high-quality source of protein for human consumption. Anim. Front..

[42] Alves R.R.N. (2009). Fauna used in popular medicine in Northeast Brazil. J. Ethnobiol. Ethnomed..

[43] Lev E. (2003). Traditional healing with animals (zootherapy): Medieval to present-day Levantine practice. J. Ethnopharmacol..

[44] Yeshi K., Morisco P., Wangchuk P. (2017). Animal-derived natural products of Sowa Rigpa medicine: Their pharmacopoeia description, current utilization and zoological identification. J. Ethnopharmacol..

[45] Tsering D., Farrington J., Norbu K. (2002). Human-wildlife conflict in the Chang Tang Region of Tibet: The impact of Tibetan brown bears and other wildlife on nomadic herds with recommendation for conflict mitigation. Series in Conservation Biology.

[46] Alves R.R.N., Pinto L.C.L., Barboza R.R.D., Souto W.M.S., Oliveira R., Vieira W. (2013). A global overview of carnivores used in traditional medicines. Animals in Traditional Folk Medicine.

[47] Reading R.P., Amgalanbaatar S., Kenny D., Onon Y., Namshir Z., De Nicola A. (2003). Argali ecology in Ikh Nartyn Chuluu nature Reserve: Preliminary findings. Mong. J. Biol. Sci..

[48] Bhatt V.P. (1999). Ethnobiology of high-altitude Himalayan communities in district Chamoli: A conservation perspective. Zoos Print J..

[49] Neelima B., Shampa J. (2015). Traditional Use of Domestic Animals among Pardhan Tribes of Chhindwara District of Madhya Pradesh, India. Int. J. Life Sci..

[50] Chen L., Liu W., Zhang Q., Xu K., Ye G., Wu W., Sun Z., Liu F., Wu K., Zhong B. (2020). RNA based mNGS approach identifies a novel human coronavirus from two individual pneumonia cases in 2019 Wuhan outbreak. Emerg. Microbes Infect..

[51] Haq S.M., Yaqoob U., Hassan M., da Silva R.J., Calixto E.S. (2020). SARS-CoV-2 Disease in North-Western Himalayan Region, India: Evolution, Forecast and Impact of Preventive Measures. Res. Square.

[52] Salafsky N., Salzer D., Stattersfield A.J., Hilton-Taylor C.R., Neugarten R., Butchart S.H., Collen B.E., Cox N., Master L.L., O’connor S.H. (2008). A standard lexicon for biodiversity conservation: Unified classifications of threats and actions. Conserv. Biol..

[53] Jacobson S.K. (2010). Effective primate conservation education: Gaps and opportunities. Am. J. Primatol..

